# Spousal Work Expectations and Psychological Distress: Insights from the United States and South Korea

**DOI:** 10.1093/geroni/igaf122.2938

**Published:** 2025-12-31

**Authors:** Linh Dang, Richard Gonzalez, Carlos Mendes de Leon, Briana Mezuk

**Affiliations:** University of Michigan, Ann Arbor, Michigan, United States; University of Michigan, Ann Arbor, Michigan, United States; Georgetown University, Washington, District of Columbia, United States; University of Michigan, Ann Arbor, Michigan, United States

## Abstract

The decision to retire or continue to work past typical retirement age often involves a joint decision-making process between spouses/partners. However, it is unclear what role, if any, spousal decision on employment influences mental health among older couples. Using the Actor-Partner Interdependence Model, this study examined the dyadic associations of work expectations with psychological distress among heterosexual older couples in the United States and South Korea. Data came from the 2016-2018 Health and Retirement Study (n = 2,009 couples) and the Korean Longitudinal Study of Aging Study (n = 385 couples). Perceived expectation of working in the next five years was reported on a probability scale (0-100%). Psychological distress was assessed by the Center for Epidemiologic Studies Depression Scale. The study found moderate to strong correlations between men and women for work expectations and psychological distress among couples. In both countries, the respondents’ own work expectations were not significantly associated with their psychological distress (OR(US) =1.01 [0.95, 1.07], OR(Korea) = 0.94 [0.68, 1.30]). There were significant spousal effects of work expectations on psychological distress among US couples (OR(US) = 1.06 [1.00, 1.11]), but no spousal effects were found among Korean couples (OR(Korea) = 0.90 [0.65, 1.24]). Moreover, the association between spousal work expectations and psychological distress did not vary by gender in either country setting. Findings emphasize the value of integrating both individual risk factors as well as contextual characteristics such as spouse/partner, gender, and country to inform mental health efforts supporting older adults during retirement transition.

